# Supplementary material for: Data-assisted reduced-order modeling of extreme events in complex dynamical systems
Source: PLoS One. 2018 May 24;13(5):e0197704. doi: 10.1371/journal.pone.0197704 (PMC5967742; doi:10.1371/journal.pone.0197704)
Supplement: S1 Appendix — In the notes we provide some background theory on recurrent neural networks, LSTM and momentum based optimization methods. Additional computation results for CDV and Kolmogorov flow are also included. (PDF) [file pone.0197704.s001.pdf]

Data-assisted reduced-order modeling of extreme events in complex dynamical systems: supplementary notes

Zhong Yi Wan[1], Pantelis Vlachas[2], Petros Koumoutsakos[2] and Themistoklis Sapsis*[1]

[1]Department of Mechanical Engineering, Massachusetts Institute of Technology, 77 Massachusetts Ave., Cambridge, MA 02139, USA
[2]Chair of Computational Science, ETH Zürich, Clausiusstrasse 33, Zürich, CH-8092, Switzerland

1 Overview of recurrent neural network and long short-term memory

1.1 Recurrent Neural Network (RNN)

RNN is a deep neural network architecture that can be viewed as a nonlinear dynamical system mapping from sequences to sequences. The most distinct feature of a RNN is the presence of hidden states whose values are dependent on those of the previous temporal steps and the current input. These units represent the internal memory of the model and are designed to address the sequential nature of the input (the same combination of inputs arranged in different order outputs different results). The output sequence is determined by the hidden state at each step. Fig S.1 shows the unfolded structure of a RNN model.

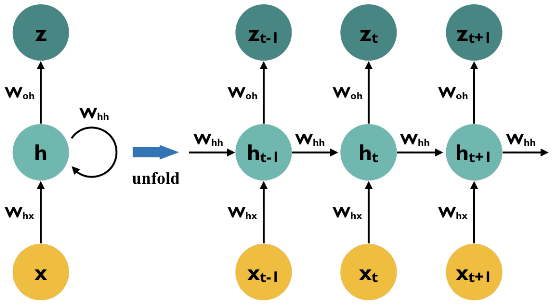

Figure S.1: Computational graph for recurrent neural network. $\mathbf{x}_t, \mathbf{h}_t$ and $\mathbf{z}_t$ are the input, hidden and output states at time t.

The model is parametrized by weight matrices $\{\mathbf{W}_{hx}, \mathbf{W}_{hh}, \mathbf{W}_{oh}\}$ and bias vectors $\{\mathbf{b}_h, \mathbf{b}_o\}$, whose concatenation are denoted by $\boldsymbol{\theta}$ for convenience. Given an input sequence $(\mathbf{x}_1, ..., \mathbf{x}_T)$,

*Corresponding author: sapsis@mit.edu, Tel: (617) 324-7508, Fax: (617) 253-8689

the RNN computes a sequence of hidden states $(\mathbf{h}_1, ..., \mathbf{h}_T)$ and a sequence of outputs $(\mathbf{z}_1, ..., \mathbf{z}_T)$ as follows

$$\begin{aligned}\mathbf{h}_t &= \sigma_h(\mathbf{W}_{hx}\mathbf{x}_t + \mathbf{W}_{hh}\mathbf{h}_{t-1} + \mathbf{b}_h)\\ \mathbf{z}_t &= \sigma_z(\mathbf{W}_{zh}\mathbf{h}_t + \mathbf{b}_z),\end{aligned} \tag{S.1}$$

where $\sigma_h(\cdot)$ and $\sigma_z(\cdot)$ are the hidden and output nonlinear activation functions respectively. The very first hidden state, $\mathbf{h}_0$, is required as an additional input to the model (or set to zero by default). Given training input and output sequences, a set of $\boldsymbol{\theta}$ can be obtained by minimizing the loss function

$$L_{\text{tot}}(\boldsymbol{\theta}) = \sum_{t=1}^{T} L\big(\mathbf{Z}_t(\boldsymbol{\theta}, \mathbf{X}), \mathbf{Y}_t\big) \tag{S.2}$$

which is a sum of per-time-step losses measuring the difference between the target output $\mathbf{Y}_t$ and model-generated $\mathbf{Z}_t$ (as a function of weights $\boldsymbol{\theta}$ and model input $\mathbf{X}$ up until time step t), which are written in upper case to emphasize that they are comprised of multiple training cases. For regression problems, the most commonly used loss function L is the mean squared error (MSE):

$$L_{\text{MSE}}(\mathbf{Z}_t, \mathbf{Y}_t) = \frac{1}{N}\sum_{n=1}^{N} \big|\big|\mathbf{z}_t^{(n)} - \mathbf{y}_t^{(n)}\big|\big|_2^2, \tag{S.3}$$

where $\mathbf{z}_t^{(n)}$ and $\mathbf{y}_t^{(n)}$ are model output and target for test case n; N is the total number of training cases. Typically, a gradient-based procedure is used for optimization, where the derivatives of L with respect to $\boldsymbol{\theta}$ are calculated using back-propagation-through-time (BPTT) algorithm [1].

1.2 Long short-term memory (LSTM)

RNNs are well known to be difficult to train in particular for problems where long-term dependencies are important. Specifically, the derivative of the loss function at one time can be exponentially large with respect to the hidden activations at an earlier time due to the nonlinear iterative nature of RNN. As a result, the gradient would either vanish or explode [2]. LSTM [3] is an effective solution for combating this problem, more specifically through introducing *memory cells*. A memory cell is a self-recurrent neuron, equipped with additional *input*, *forget* and *output gates*. The presence of these gates allows control over, depending on the input and the previous hidden state, how much memory is received and passed on to the next time-step, as well as how much to take from the input. When the gates are shut, the gradients can flow through the memory unit without alteration for an indefinite amount of time, thus overcoming the vanishing gradient problem.

Fig S.2 shows the unrolled structure of a LSTM model. The evolution of the states are governed by the following equations:

$$\begin{aligned}\mathbf{i}_t &= \sigma(\mathbf{W}_{ih}\mathbf{h}_{t-1} + \mathbf{W}_{ix}\mathbf{x}_t + \mathbf{b}_i)\\ \mathbf{f}_t &= \sigma(\mathbf{W}_{fh}\mathbf{h}_{t-1} + \mathbf{W}_{fx}\mathbf{x}_t + \mathbf{b}_f)\\ \mathbf{o}_t &= \sigma(\mathbf{W}_{oh}\mathbf{h}_t + \mathbf{W}_{ox}\mathbf{x}_t + \mathbf{b}_o)\\ \tilde{\mathbf{C}}_t &= \tanh(\mathbf{W}_{ch}\mathbf{h}_{t-1} + \mathbf{W}_{cx}\mathbf{x}_t + \mathbf{b}_c)\\ \mathbf{C}_t &= \mathbf{i}_t \circ \tilde{\mathbf{C}}_t + \mathbf{f}_t \circ \mathbf{C}_{t-1}\\ \mathbf{h}_t &= \mathbf{o}_t \circ \tanh(\mathbf{C}_t),\end{aligned} \tag{S.4}$$

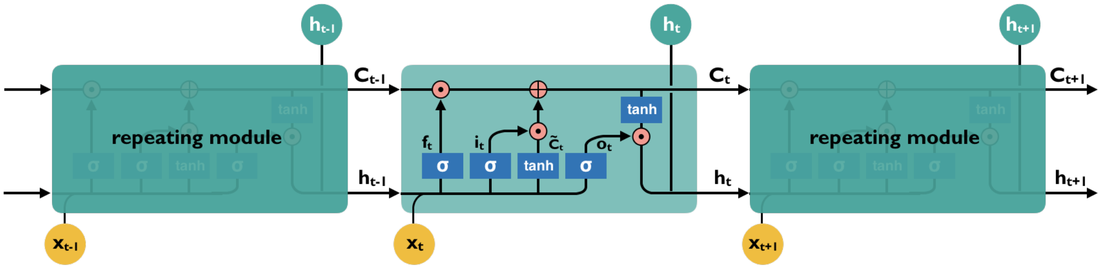

Figure S.2: The repeating module in an LSTM. $\mathbf{x}_t$ and $\mathbf{h}_t$ are input and output states at time t; $\mathbf{C}_t$ is the running memory; $\mathbf{f}_t$, $\mathbf{i}_t$ and $\mathbf{o}_t$ are forget, input and output gate values calculated based on previous output $\mathbf{h}_{t-1}$ and current input $\mathbf{x}_t$; $\tilde{\mathbf{C}}_t$ is a candidate memory state that is integrated with the previous memory to generate current memory $\mathbf{C}_t$

where $\sigma(\cdot)$ is the sigmoid function and $\circ$ denotes element-wise multiplication. $\mathbf{i}_t, \mathbf{f}_t$ and $\mathbf{o}_t$ are the input, forget and output gates respectively. $\mathbf{C}_t$ represents the cell memory (formally called *cell state*) calculated as a linear combination of a new candidate cell state $\tilde{\mathbf{C}}_t$ and the previous memory $\mathbf{C}_{t-1}$, weighted by the input and forget gate values. $\mathbf{h}_t$ is the output of the LSTM and can be regarded as a nonlinear copy of the cell state that helps to decide how to process the input in the next step. All gates, cell states and outputs have the same dimension n_{LSTM} which is a hyperparameter of the model. It determines the memory capacity of the network and is in general different from the target dimension. Hence, $\mathbf{h}_t$ often has to connect to additional fully-connected layers to arrive at the desired dimension in the final output $\mathbf{z}_t$, such as the architectures used in this work.

In total, 8 weight matrices and 4 bias vectors make up the parameter space of a single LSTM unit. Despite having more parameters and a much more intricate forward pass, LSTM can be easily implemented using software packages equipped with automatic differentiation. In this work, all models are implemented in Keras [4] with a Tensorflow [5] backend.

1.3 Weight optimization

Here we provide a brief description of the algorithm used for weight optimization in this work. We start by noting that the total loss function (MSE based) can be rewritten as a sum of individual losses over test cases:

$$L_{\text{tot}}(\boldsymbol{\theta}) = \frac{1}{N}\sum_{n=1}^{N}\sum_{t=1}^{T}||\mathbf{z}_t^{(n)} - \mathbf{y}_t^{(n)}||_2^2 \equiv \frac{1}{N}\sum_{n=1}^{N} L_{\text{MSE}}^{(n)}(\boldsymbol{\theta}|\mathbf{X}, \mathbf{Y}). \tag{S.5}$$

where $L_{\text{MSE}}^{(n)}$ denotes the loss for the nth training trajectory over all time steps. A standard gradient descent method performs the following update

$$\boldsymbol{\theta} := \boldsymbol{\theta} - \nabla_{\boldsymbol{\theta}} L_{\text{tot}} = \boldsymbol{\theta} - \eta \sum_{n=1}^{N} \nabla_{\boldsymbol{\theta}} L_{\text{MSE}}^{(n)}/N, \tag{S.6}$$

where η is a step size more commonly known as the *learning rate*. The gradient $\nabla_{\boldsymbol{\theta}} L_{\text{tot}}$, similar to the loss function itself, is expressed as a sum over a gradient function evaluated on individual test cases. Computing this gradient in practice can be very slow and memory-intensive for large data sets. To this end, we often resort to a mini-batch gradient descent algorithm:

$$\boldsymbol{\theta} := \boldsymbol{\theta} - \eta \sum_{n=n_1}^{n_2} \nabla_{\boldsymbol{\theta}} L_{\text{MSE}}^{(n)}/N, \tag{S.7}$$

where $n_{\text{batch}} = n_2 - n_1$ is a fixed batch size hyperparameter and n_1 is iterated through the training set at n_{batch} intervals to ensure that all training examples are covered. In this way, gradient is updated based on *mini-batches* of training examples, which serves to (a) reduce the variance of the parameter updates for more stable convergence and (b) make the process more memory-efficient. This approach is known as *stochastic gradient descent* (SGD) and is the foundation for most of the optimization algorithms used for neural networks today.

Choosing an appropriate learning rate η schedule, however, remains difficult. A learning rate that is too small leads to slow convergence while a large learning rate may potentially cause fluctuations near the optimum or even divergence. In an attempt to tackle this issue, the concept of *momentum* [6] is introduced that helps accelerate SGD in relevant directions and dampens potential oscillations. This is accomplished by adding a fraction α of the cumulative update vector to the current gradient:

$$\begin{aligned} \boldsymbol{\nu}_t &:= \alpha \boldsymbol{\nu}_{t-1} + \eta \nabla_{\boldsymbol{\theta}} L \\ \boldsymbol{\theta} &:= \boldsymbol{\theta} - \boldsymbol{\nu}_t, \end{aligned} \tag{S.8}$$

where t is the index of the current update. This momentum term automatically increases the update in dimensions whose recent gradients point in similar directions and decreases those for dimensions whose gradients change directions, thereby gaining faster convergence and reducing oscillations.

Building on this concept, the adaptive moment estimation (Adam) algorithm [7] applies additional levels of momentum control:

$$\begin{aligned} \boldsymbol{\nu}_t &:= \beta_1 \boldsymbol{\nu}_{t-1} + (1-\beta_1)\nabla_{\boldsymbol{\theta}} L \\ \boldsymbol{m}_t &:= \beta_2 \boldsymbol{m}_{t-1} + (1-\beta_2)(\nabla_{\boldsymbol{\theta}} L)^2 \\ \hat{\boldsymbol{\nu}}_t &= \boldsymbol{\nu}_t/(1-\beta_1^t) \\ \hat{\boldsymbol{m}}_t &= \boldsymbol{m}_t/(1-\beta_2^t) \\ \boldsymbol{\theta} &:= \boldsymbol{\theta} - \frac{\eta}{\sqrt{\hat{\boldsymbol{m}}_t} + \epsilon} \circ \hat{\boldsymbol{\nu}}_t, \end{aligned} \tag{S.9}$$

where ϵ is a smoothing constant that helps avoid division by zero. Here $\boldsymbol{\nu}$ and $\boldsymbol{m}$ are (weighted) history of the gradient and its squared value obtained in the past. The division of $1-\beta_1^t$ and $1-\beta_2^t$ counteracts the bias resulted from zero initializations to $\boldsymbol{\nu}$ and $\boldsymbol{m}$. Performing a scalar division by $\hat{\boldsymbol{m}}_t$ on the update dampens any extreme updates experienced previously. This method has seen great success in practice and utilized for optimizing the model architectures presented in this work.

2 The Charney DeVore system

2.1 Proper orthogonal decomposition

In this section, we describe the proper orthogonal decomposition (POD) applied to the Charney-DeVore system. For a set of snapshot state vectors $\{\mathbf{x}^{(n)}\}_{n=1}^N$ in $\mathbb{R}^d$ (centered to zero mean), POD seeks an orthogonal projector $\mathbf{\Pi_V}$ of fixed rank m that minimizes the projection error

$$\sum_{n=1}^{N} ||\mathbf{x}^{(n)} - \mathbf{\Pi_V}\mathbf{x}^{(n)}||^2 = J(\mathbf{\Pi_V}), \tag{S.10}$$

where $\mathbf{\Pi_V}\mathbf{x}^{(n)} = \mathbf{V}\mathbf{V}^T\mathbf{x}^{(n)}$ and columns of $\mathbf{V}$ form an orthonormal basis for an m-dimensional subspace of $\mathbb{R}^d$. One can show that the optimal basis is given by the leading eigenvectors of the empirical covariance matrix

$$\mathbf{C} = \frac{1}{N}\sum_{n=1}^{N} \mathbf{x}^{(n)}(\mathbf{x}^{(n)})^T. \tag{S.11}$$

We denote the leading eigenvalues and normalized leading eigenvectors by $\{\lambda_1, \ldots, \lambda_m\}$ and $\{\mathbf{v}_1, \ldots, \mathbf{v}_m\}$. This projection has the property that the new coordinates $\xi_i = \mathbf{v}_i^T\mathbf{x}, i = 1, \ldots, m$ are uncorrelated. In addition, the eigenvalue λ_i represents the variance of the projected coordinate (i.e. var$[\xi_i]$) and for full-dimensional projection $m = d$ the sum of eigenvalues is equal to the total variance summed over the dimensions of $\mathbf{x}$. The eigenvalues and percentage variance of each mode for the CDV system, computed from the $N = 10^4$ data set, is listed in Table S.1.

Mode number	Mode variance	Cumulative variance (%)
1	4.7×10^{-2}	66.5
2	1.9×10^{-2}	93.2
3	3.2×10^{-3}	97.6
4	9.1×10^{-4}	98.9
5	5.2×10^{-4}	99.6
6	2.8×10^{-4}	100.0

Table S.1: POD variance spectrum for CDV system

2.2 Additional computational results

In this section we present additional numerical results for using the proposed data-driven approach to assist the POD-projected model in lower-dimensional subspaces (dimensions 3 and 4). The architectures used in each case have the same number of hidden units in the LSTM layer as the number of *truncated* dimensions. Similar to the 5-dimensional reduced-order model, presented in the main text, we use architecture I and perform training for 1000 epochs. The other hyperparameters remain unchanged. Fully data-driven models are also constructed and tested for comparison.

In Figs S.3 and S.4 we show the model performance in terms of RMSE, ACC and the two sample trajectories used in Fig 4 for 4- and 3-dimensional subspaces respectively. As expected, the error for the lower dimensional data-assisted model is in general larger and the short-term accuracy comes closer to that of the fully data-driven model. This result demonstrates that there is tremendous value to a good reduced-order model for which the assistive data-driven strategy is used, as it helps to greatly reduce the difficulties of learning long-term dependencies from data due to the presence of chaos.

Fig S.5 shows the attractor of each data-assisted model compared with that of the true system (projected to first two POD dimensions). For both 4- and 3-dimensional reduced-order model, the data-assisted strategy is able to reconstruct a strange attractor despite the fact that the projected equations both have a single fixed point. This means that the equation-based dynamics is sufficient to warrant long term stability (the corresponding fully data-driven models diverge). However, the quality at which the resulted attractor replicates that of the full system clearly deteriorates as the reduction dimension lowers.

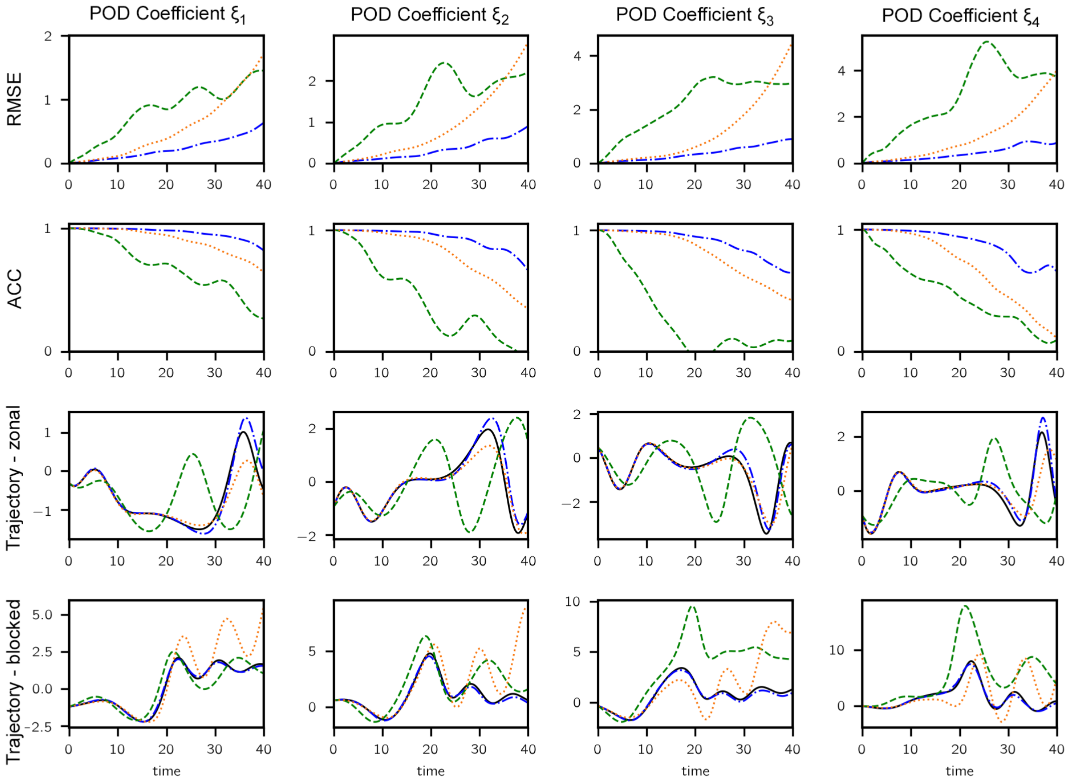

Figure S.3: RMSE vs. lead time (first row), ACC vs. lead time (second row) and two example trajectories (last two rows) comparing the performance of data-assisted approach (-·-), fully data-driven model (- -) and projected equations alone (·····) for a *4-dimensional* reduced-order model based on the most energetic POD modes. For the test trajectories, truth (—) is also included. All values are normalized by the square root of the POD eigenvalues.

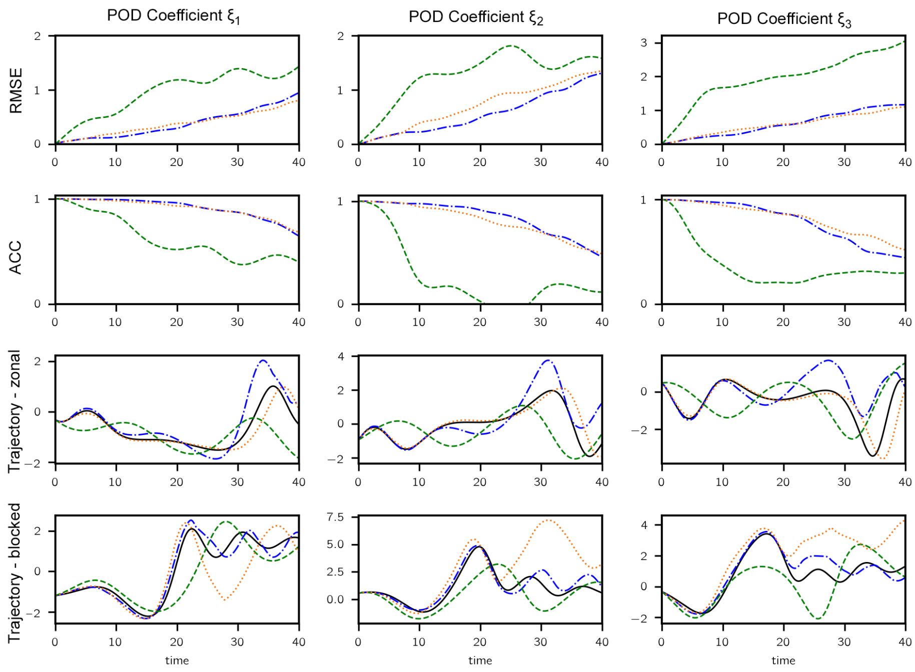

Figure S.4: RMSE vs. lead time (first row), ACC vs. lead time (second row) and two sample trajectories (last two rows) comparing the performance of data-assisted approach (-·-), fully data-driven model (- -) and projected equations alone (···) for a *3-dimensional* reduced-order model based on the most energetic modes. For the test trajectories, truth (—) is also included. All values are normalized by the square root of the POD eigenvalues.

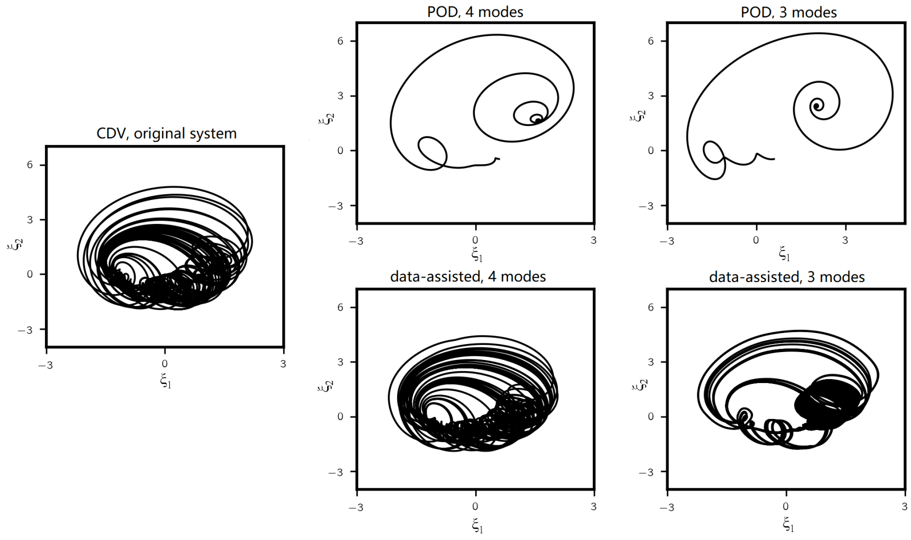

Figure S.5: 2000-unit trajectories projected to the (ξ_1, ξ_2) plane for 4- and 3-dimensional projected and data-assisted models. The projected equation-based models result in a single fixed point while data-assisted models are able to replicate the true system attractor much better.

3 The Navier-Stokes equation

3.1 Triad dynamics

We provide a derivation for triad dynamics (20). Starting from the original model equations (17), one can show that ∇p is orthogonal to *all* divergence-free vector fields $\{\mathbf{v} : \nabla \cdot \mathbf{v} = 0\}$ and can therefore be interpreted as taking an orthogonal projection onto the subspace. The pseudo-differential operation is known as the *Leray projection.* Hence we can remove the pressure field dependence and rewrite (17) as

$$\partial_t \mathbf{u} = -\mathbf{P}[\mathbf{u} \cdot \nabla \mathbf{u}] + \nu \Delta \mathbf{u} + \mathbf{f} \tag{S.12}$$

where $\mathbf{P}$ denotes the Leray projection operator. Substituting in the Fourier coefficients defined in (19), components of the nonlinear term $\mathbf{u} \cdot \nabla \mathbf{u}$ can be expressed explicitly as

$$\begin{aligned} u_x \frac{\partial u_x}{\partial x} + u_y \frac{\partial u_x}{\partial y} &= i \sum_{\mathbf{p}} \sum_{\mathbf{q}} \frac{a(\mathbf{p}) a(\mathbf{q})}{|\mathbf{p}||\mathbf{q}|} (p_2 q_1 q_2 - p_1 q_2^2) e^{i(\mathbf{p}+\mathbf{q})\cdot \mathbf{x}} \\ u_x \frac{\partial u_y}{\partial x} + u_y \frac{\partial u_y}{\partial y} &= i \sum_{\mathbf{p}} \sum_{\mathbf{q}} \frac{a(\mathbf{p}) a(\mathbf{q})}{|\mathbf{p}||\mathbf{q}|} (p_1 q_1 q_2 - p_2 q_1^2) e^{i(\mathbf{p}+\mathbf{q})\cdot \mathbf{x}} \end{aligned} \tag{S.13}$$

In 2D, the projection operator takes the form

$$\mathbf{P} = \frac{1}{|\mathbf{k}|^2} \begin{bmatrix} k_2^2 & -k_1 k_2 \\ -k_1 k_2 & k_1^2 \end{bmatrix}. \tag{S.14}$$

Substituting (S.13) and (S.14) into (S.12), we obtain the evolution equation in Fourier space

$$\begin{aligned} \partial_t \hat{u}_x(\mathbf{k}) =& -i \sum_{\mathbf{p}+\mathbf{q}=\mathbf{k}} [k_2^2 q_2 (p_2 q_1 - p_1 q_2) - k_1 k_2 q_1 (p_1 q_2 - p_2 q_1)] \frac{a(\mathbf{p}) a(\mathbf{q})}{|\mathbf{p}||\mathbf{q}||\mathbf{k}|^2} \\ & - \nu |\mathbf{k}|^2 \hat{u}_x(\mathbf{k}) - \frac{1}{2} i \left(\delta_{\mathbf{k},\mathbf{k}_f} - \delta_{\mathbf{k},-\mathbf{k}_f} \right) \\ \partial_t \hat{u}_y(\mathbf{k}) =& -i \sum_{\mathbf{p}+\mathbf{q}=\mathbf{k}} [k_1^2 q_1 (p_1 q_2 - p_2 q_1) - k_1 k_2 q_2 (p_2 q_1 - p_1 q_2)] \frac{a(\mathbf{p}) a(\mathbf{q})}{|\mathbf{p}||\mathbf{q}||\mathbf{k}|^2} \\ & - \nu |\mathbf{k}|^2 \hat{u}_y(\mathbf{k}), \end{aligned} \tag{S.15}$$

which is identical to (20).

3.2 Initial transient effects

In our framework we have expressed the complementary dynamics as a function of past states in the reduction space. However, we must start the forecast at some initial condition where no history is available and the model needs to read a few input states in order to “catch up to speed”. During this transient stage, predictions are not expected to be accurate. It is of great interest to us to estimate the length of this transient stage so that low weights can be placed on the corresponding errors. More importantly, such an estimate provides an idea regarding the minimum set-up stage length s for architecture II.

For the Navier-Stokes equation presented in this work, this parameter is determined empirically: we make a guess, perform training on architecture I and modify guessed value based on validation results. In Fig S.6 we provide the prediction validation plot for an example case showing the predicted and true values of the complementary dynamics at each step of a trained

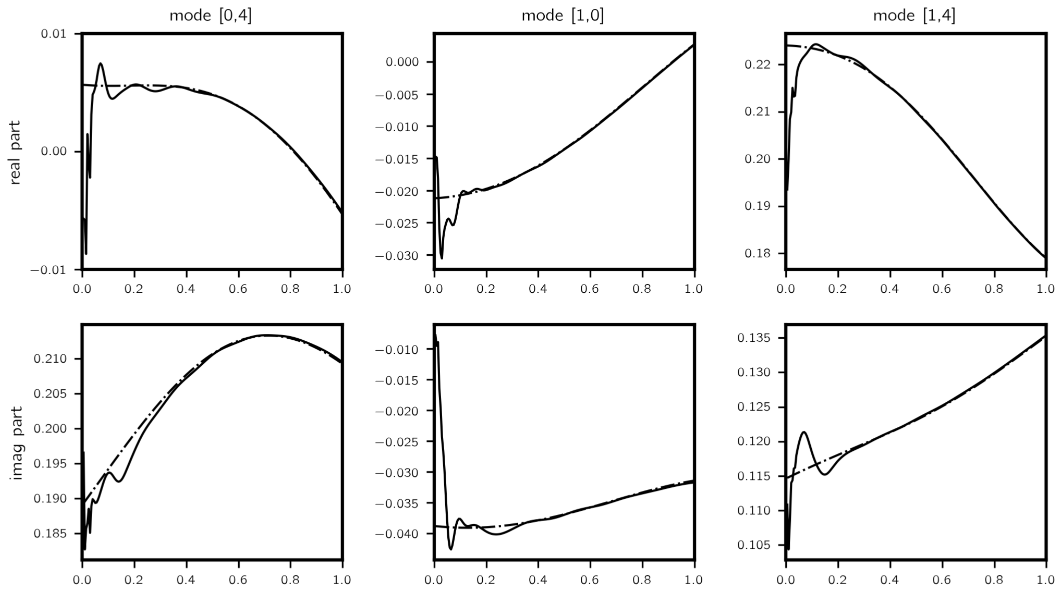

Figure S.6: Kolmogorov flow: prediction for a validation case using architecture I ($p = 200$, $\tau = 0.005$, $n_{\text{LSTM}} = 70$ and $n_{\text{FC}} = 38$). Solid and dashdotted lines are predicted and true complementary dynamics respectively. Horizontal axis is time. Predictions before time 0.4 are not expected to be accurate because model memory is initialized to zero.

model. As expected, oscillations and low accuracy are observed for the first few steps. However, predictions soon converge almost perfectly onto the true values as the effects of zero initialization to the model memory gradually die out. Moreover, the time it takes for oscillations to fade differs across dimensions and it makes most sense to use the longest. We use $p_t = 60$ and a much more conservative $s = 100$ for the final results. Coincidentally, the length of the set-up sequence $s\tau = 0.5$ agrees approximately with the eddy turn-over time of the system.

3.3 Sequential training with increasing p

As discussed in the main text, we progressively improve the model weights by training a sequence of models starting from architecture I and gradually increasing the length of the prediction stage of architecture II. Smaller learning rates have to be used to for models with longer prediction stage in order to achieve stable and steady improvement in training loss. The model structure and associated learning rate used is listed in Table S.2. This training profile is found to steadily reduce the multi-step prediction error of this problem. The test RMSE of the weights obtained at the end of each training step is shown in Fig S.7. During each training step the weights are optimized for 1000 epochs.

Model	learning rate η
arch I	1E-3
arch II, p = 10	1E-3
arch II, p = 30	5E-4
arch II, p = 50	1E-4
arch II, p = 100	1E-5

Table S.2: Learning rate for sequential model training. Model with a larger p is more sensitive to the weights due to chaos and thus smaller learning rate has to be used.

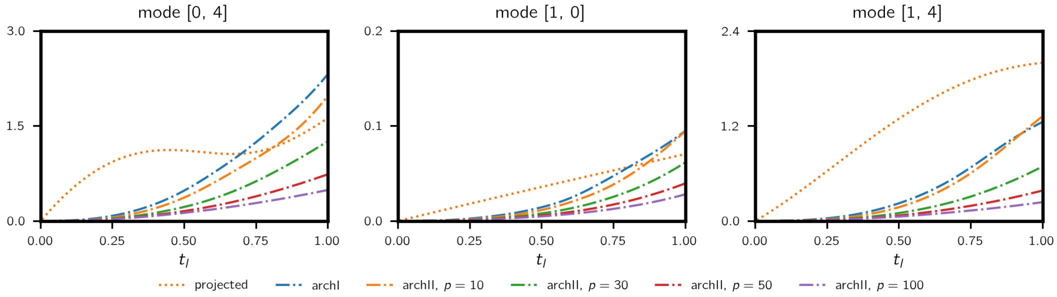

Figure S.7: Test RMSE for weights obtained from architecture I and architecture II at $p = \{10, 30, 50, 100\}$. At each step the model is trained for 1000 epochs at decreasing learning rate. Error performance is steadily improved.

References

[1] P. Werbos. Backpropagation through time: what it does and how to do it. *Proc. IEEE*, 78(10):1550–1560, 1990.

[2] Y. Bengio, P. Simard, and P. Frasconi. Learning long-term dependencies with gradient descent is difficult. *IEEE Trans. Neural Networks*, 5(2):157–166, March 1994.

[3] Hochreiter, Sepp and Schmidhueber, Juergen. Long short-term memory. *Neural Computation*, 9(8):1–32, 1997.

[4] F. Chollet et al. Keras. `https://github.com/fchollet/keras`, 2015.

[5] M. Abadi et al. Tensorflow: Large-scale machine learning on heterogeneous systems. `https://www.tensorflow.org/`, 2015.

[6] D. E. Rumelhart, G. E. Hinton, and R. J. Williams. Learning representations by back-propagating errors. *Nature*, 323(6088):533–536, 1986.

[7] D.P. Kingma and J. Ba. Adam: A method for stochastic optimization. *CoRR*, abs/1412.6980, 2014.
